# Supplementary material for: Mechanisms governing the pioneering and redistribution capabilities of the non-classical pioneer PU.1
Source: Nat Commun. 2020 Jan 21;11:402. doi: 10.1038/s41467-019-13960-2 (PMC6972792; doi:10.1038/s41467-019-13960-2)
Supplement: Supplementary file 7 — Source data [file 41467_2019_13960_MOESM7_ESM.zip › Source_Data/FigureS2/FigureS2B_MotifScanOutput/KmeansCluster14/homerResults/motif14.similar.html]

motif14

## Information for motif14

A
G
C
T
G
A
C
T
A
G
T
C
T
A
G
C
C
A
T
G
A
G
T
C
A
T
G
C
G
A
T
C
A
G
T
C
A
G
C
T
G
A
C
T
A
G
C
T
A
G
T
C
  
Reverse Opposite:  

C
T
A
G
C
T
G
A
C
T
G
A
C
T
G
A
A
C
T
G
C
T
A
G
A
T
C
G
A
C
T
G
G
T
A
C
A
T
C
G
A
C
T
G
C
T
G
A
T
C
G
A
  

|  |  |
| --- | --- |
| p-value: | 1e-17 |
| log p-value: | -4.079e+01 |
| Information Content per bp: | 1.769 |
| Number of Target Sequences with motif | 29.0 |
| Percentage of Target Sequences with motif | 5.69% |
| Number of Background Sequences with motif | 277.0 |
| Percentage of Background Sequences with motif | 0.61% |
| Average Position of motif in Targets | 90.5 +/- 52.3bp |
| Average Position of motif in Background | 101.9 +/- 78.1bp |
| Strand Bias (log2 ratio + to - strand density) | -0.5 |
| Multiplicity (# of sites on avg that occur together) | 1.08 |
| Motif File: | file (matrix) reverse opposite |

### Similar de novo motifs found

|  |  |  |  |  |  |  |  |
| --- | --- | --- | --- | --- | --- | --- | --- |
| Rank | Match Score | Redundant Motif | P-value | log P-value | % of Targets | % of Background | Motif file |
| 1 | 0.941 | T A C G T C A G C T G A C G T A C T G A C T A G T C A G T C A G C T A G G T A C A T C G A C T G T C G A T G C A | 1e-17 | -40.038913 | 4.12% | 0.25% | motif file (matrix) |
| 2 | 0.931 | T A G C T G A C C T A G T G A C T G A C G A T C G A T C G A C T G A C T A G C T A T G C | 1e-14 | -33.334188 | 8.82% | 2.11% | motif file (matrix) |
| 3 | 0.861 | C G T A C T G A C T A G A C T G T A C G A T C G T G A C C A T G A C T G C G T A T C G A G C T A | 1e-13 | -32.214575 | 5.88% | 0.94% | motif file (matrix) |
| 4 | 0.850 | C G T A C G A T T G C A C A T G T C A G T C A G T A C G T A G C T A C G A T C G | 1e-12 | -27.910475 | 5.69% | 1.04% | motif file (matrix) |
